# Supplementary material for: “Everything was much more dynamic”: Temporality of health system responses to Covid-19 in Colombia
Source: PLoS One. 2024 Sep 26;19(9):e0311023. doi: 10.1371/journal.pone.0311023 (PMC11426449; doi:10.1371/journal.pone.0311023)
Supplement: S1 Table — (PDF) [file pone.0311023.s002.pdf]

S1 Table. Interview Participants

Code	Description	City	Level	Year
**Laboratories**				
SH-D-011	Laboratory representative	Cartagena	Local	2020
SH-A-018	National health agency representative	Bogotá	National	2020
SH-D-24	University director	Cartagena	Local	2020
SH-B-30	University academic	Bogotá	Local	2020
SH-C-025	Laboratory representative	Cali	Local	2020
SH-D-025	Laboratory representative	Cartagena	Local	2020
SH-C-024	Laboratory director	Cali	Local	2020
**Intensive Care Units**				
SH-B-018	Representative, commissioning organization	Bogotá	Local	2020
SH-A-001	Nursing association representative	Bogotá	National	2020
SH-A-005	Physician, non-governmental organization	Bogotá	Local	2020
SH-A-007	National research coordinator	Bogotá	National	2020
ES-B-003	Hospital director	Bogotá	Local	2020
SH-D-028	Hospital physician	Cartagena	Local	2020
SH-C-021	Hospital nurse	Cali	Local	2020
SH-A-018	National health agency representative	Bogotá	National	2020
