# Supplementary material for: “Everything was much more dynamic”: Temporality of health system responses to Covid-19 in Colombia
Source: PLoS One. 2024 Sep 26;19(9):e0311023. doi: 10.1371/journal.pone.0311023 (PMC11426449; doi:10.1371/journal.pone.0311023)
Supplement: S2 Table — (PDF) [file pone.0311023.s003.pdf]

S3 Table. Coding Tree

Theme category	Subcategories	Task	
		Labs	ICUs
1. Enacting temporal urgency	Demanding rapid adaptation	X	X
	Motivational variety	X	X
2. Enacting historical learning	Relevant of past experiences and skills	X	X
	Rekindling of latent relationships	X	X
	Perceived system failings historically	X	X
3. Evaluating consequences of the approach to change	Pace of implementation	X	X
	Concerns about staff wellbeing	X	X
	Missteps in task navigation	X	X
	Resource bottlenecks	X	
	Administrative capacity to make adaptations	X	
	Lack of staff involvement in decision-making		X
	Technical issues with information systems		X
	Time pressures on staff		X
	Financial cashflow		X
	Speeding up internal management processes		X
