# Supplementary material for: “Everything was much more dynamic”: Temporality of health system responses to Covid-19 in Colombia
Source: PLoS One. 2024 Sep 26;19(9):e0311023. doi: 10.1371/journal.pone.0311023 (PMC11426449; doi:10.1371/journal.pone.0311023)
Supplement: S3 Table — (PDF) [file pone.0311023.s004.pdf]

S4 Table. Laboratories Case Narrative

Problem

In March 2020, the need to "test, test, test" was proclaimed by the head of the World Health Organization to trace and mitigate the spread of COVID-19 within and among nations [36]. In Colombia, the process of testing was initially coordinated and delivered at national level by the National Institute for Health. However, the centralized approach drawing on existing facilities was rapidly overwhelmed by the volume of samples submitted, resulting in the delay of test results beyond any useful period, as this interviewee referring to the situation in Cartagena highlighted:

"the norm says that you have to have an answer within 48 hours, and we started to have an answer very late with the institute, two days, three days, four days, five days, one week, ten days, 15 days, 20 days... and you know that a result of a COVID at 20 days is already for what [purpose], so it doesn't make sense; but it wasn't anyone's fault, it was that the institute received 8,000, 10,000 and 12,000 samples a day." (SH-D-11, Laboratory representative, Cartagena).

Given the scaling of testing required to respond to the pandemic – both in geographical spread and by volume - the need to expand and decentralize laboratory testing facilities was recognized during the early months of the first wave. To support the expansion of Colombia´s national testing infrastructure, laboratories were invited to seek accreditation to provide additional testing services, with the aim of improving the timely diagnosis and tracing of positive cases. The certification of laboratories required coordination among a series of stakeholders at local and national level: between the laboratory leads and wider organizations (e.g. universities) in which they were situated; local authorities, including health secretariats and mayor´s offices, who were involved in the supply of funding and key resources (e.g. biosecurity equipment, reagents and lab pipette tips); and leadership from national entities including the national institute for health, national health observatory, and ministry of health.

Actions

Key tasks for the laboratories included hiring and training new staff, accumulating resources for performing the tests, and securing funding to repurpose and run the laboratories. As well as formal relationships among the stakeholders described, laboratory leads relied reportedly on informal practices and relationships to navigate the accreditation process and service the testing of samples (especially where there were delays in the supply of resources through the formal relationships). First, informal collaboration among stakeholder entity leads – stimulated by shared ties stemming from being practising researchers in similar disciplinary fields – underpinned initial responses to the pandemic. For instance, a social messaging group was formed by two research collaborators linked to a national health entity in mid-January prior to the pandemic reaching Colombia, based on recognition of the need for

preparatory learning at that time: “we are days away from [Covid-19] arriving” (SH-A-018, national health agency representative, Bogotá).

Second, with regard to funding the programme of repurposing laboratories, we were told that “informal” sources of funding, as well as voluntary donations, helped to address delays to, or the absence of, the allocation of formal funding at state level to expand the testing infrastructure. For example, a representative from the national entity overseeing the expansion of testing, suggested that their entity´s historic research links to an Anglo-American network through collaborating on the response to previous infectious disease outbreaks, had provided early access to funding while “formal” national level sources in Colombia were still being awaited.

Third, the need to safeguard staff involved in scaling up the testing capacity was described to us. Due to the increased intensity of work associated with pandemic working (e.g. some staff took on additional COVID-19 roles while maintaining their existing responsibilities), workforce “slack” was addressed through monetary and non-monetary adjustments to staff working conditions, which ranged from the payment of overtime hours for staff on integrated contracts (where typically the contract subsumed variations in labour time) and the introduction of ad hoc space to support extended periods of working (e.g. a makeshift gym including basic sleeping arrangements was set up at the workplace).

Provisional outcomes

The scaling up of laboratory testing resulted in the commissioning of a mixed economy of 110 laboratories. Of the laboratory facilities nationally, 18.51% (30 labs) were represented by academic molecular biology laboratories. In March 2020, the month of COVID-19´s reported arrival in Colombia, there was an average of 639 PCR test samples processed per day; by late July 2020, this had risen to 24,733 samples [37]. As the national network of accredited laboratories expanded, the national centre was able to take on a network governance role, including monitoring quality assurance and brokering information sharing agreements.
