# Supplementary material for: “Everything was much more dynamic”: Temporality of health system responses to Covid-19 in Colombia
Source: PLoS One. 2024 Sep 26;19(9):e0311023. doi: 10.1371/journal.pone.0311023 (PMC11426449; doi:10.1371/journal.pone.0311023)
Supplement: S4 Table — (PDF) [file pone.0311023.s005.pdf]

S5 Table. Consequences of accelerated approach to scaling up laboratory capacity.

Impact	Evidence
Pace of implementation	Overcoming “resistance” to change:<br><br>“They have been clear about this for a long time, in fact, here the board of directors at the university, people have always told them that they are crazy […] when they say, "we want to do this, such a thing, so, so", there is always resistance at the lower levels, at the lower levels there is always resistance, always: ´no, no, no, that can't be done, it can't be done, it can't be done, that's very difficult, no´. It is always very negative the matter, so they stick out their chest and say: “see, it can be done?” (SH-D-24, university director, Cartagena).<br><br>Absorption of additional roles and responsibilities:<br><br>“Those two are impressive. I have many people to thank but they [both] have taken on completely different roles […] they have not told me, "oh, take away my blood bank job", not at all, they continue to do tremendous tasks. But they also have new roles that the [agency] did not have” (SH-A-018, national health agency representative, Bogotá).
Staff wellbeing	Fulfilling unusually long work shifts:<br><br>“it was seven continuous weeks, that team working every day, without a single day of rest, about 14, 15 hours a day, we didn't have any more staff and there were days when we had to work 24 hours a day and there were days when we had to do 36 hour shifts because there was no way, there was no one to work with and we had the lab collapsed [...] The four people who came to the university who saw us in the corridors said, "you are finished, you are zombies, you look like a vampire, you, what happened to you". We were totally… we lost weight, the dark circles under our eyes, the tiredness, but there was no other option until we managed to

	train the bacteriologists” (SH-D-24, university director, Cartagena).
Missteps in the process	Resource bottlenecks:<br><br>“the coronavirus is on one side, in one corner of the ring and we, the rest of humanity are in the other, united and in unison, doing our job. And that in theory should be so. I think that at the beginning, and even now, everyone says, "yes, yes, hello, we have to work together". But apart from the disarticulation of administration that I described a moment ago, there is also the fact that resources are not flowing. The payment for the services for which they are doing the services is not coming” (SH-C-025, Laboratory representative, Cali).<br><br>“We had to buy printers, computers, we had to expand the internet because the internet was falling down because it was so much information; in other words, everything caught us with our hands down, just like they caught everyone in Colombia, and everyone in the world. I am almost sure that if we do an interview with a laboratory in Italy, the same thing happened; and from Spain, the same story; that is to say, here nobody was prepared for this pandemic situation and that in one way or another it is a situation that is out of the ordinary and not easy to handle.” (SH-D-011, Laboratory representative, Cartagena).
