# Supplementary material for: “Everything was much more dynamic”: Temporality of health system responses to Covid-19 in Colombia
Source: PLoS One. 2024 Sep 26;19(9):e0311023. doi: 10.1371/journal.pone.0311023 (PMC11426449; doi:10.1371/journal.pone.0311023)
Supplement: S5 Table — (PDF) [file pone.0311023.s006.pdf]

S6 Table. ICU Case Narrative

Problem

ICU capacity needed to be adapted and expanded to meet the needs of Covid-19 patients following the pandemic´s arrival in Colombia in early March 2020. To support the health system´s response to Covid-19, a series of legal decrees were introduced by national government. Key among these was decree 538 [38], of 12 April 2020, which included several features that influenced the management of ICUs and hospital biosecurity: (1) expand ICU capacity for treating Covid-19 and other intensive care patients by aiming to increase the number of ICU beds from 5,349 to 15,596; (2) centralize the management of ICUs via the Regulatory Centres of Urgencies, Emergencies and Disasters (*CRUE*, in Spanish); (3) facilitate providers to set, transform and/or expand their health services rapidly with authorization by local health commissioners; (4) to provide services outside the hospital setting, including use of telemedicine, with local commissioners´ approval; (5) recognize Covid-19 as an occupational disease (following WHO guidance) and make additional economic payments to staff in health service-related roles as necessary; (6) allow the contracting of, and sharing of resources among, public and privately owned service providers; and (7) make advance payments as necessary to both public and privately owned providers to expand ICU capacity, including investments in biosecurity measures.

Actions

According to the World Health Organization´s living guideline on clinical management of Covid-19 patients [39], when a patient accesses the health system, care involves five stages: screening, triage, clinical assessment, treatment, and release. The recommended pathway emphasizes safety measures for controlling infectious disease, including isolation of patients and use of personal protective equipment (PPE). For providers involved in the expansion of ICU facilities in Colombia, actions necessary included: (1) gain authorization from local health care commissioners, (2) ensure the safety of care delivery, including implementation of isolation and PPE measures, (3) coordinate service delivery with external agencies, including CRUE, and adjacent providers for either the public or private sector, (4) secure access to sources of finance and clinical resources, (5) scale up and protect the workforce associated with ICUs, and (6) deliver effective treatment in accordance with clinical guidance.

Provisional outcomes

With regard to ICU capacity, the number of beds peaked at 13,120 in June 2021, falling back to 8,446 by October 2022 [40]; at no point did demand for beds outstrip capacity nationally, although ICU occupancy reportedly reached 97% in Bogotá in June 2021, coinciding curiously with the announcement of the relaxation of lockdown measures at this time (and "red alerts" concerning bed occupancy levels were made at local level periodically during the pandemic) [41]. Our stakeholder interviews highlighted perceived challenges with adapting and scaling up ICUs, including resourcing and financial flows:

"We could say that there are deficits in terms of infrastructure. We do have a shortage of personnel, but we also have very few staff trained in intensive care units, and we have a deficit in intensive care units of elements for respiratory patient care" (SH-A-001, nursing association representative, Bogotá).

“we have a human resource contracted and defined to attend a significant volume of patients, and now we have an expectant human resource, with a certain cost, but without generating care processes and if we do not generate care processes, there is no income that can give us the support for the economic sustainability of the services” (SH-A-005, physician, non-governmental organization, Bogotá).

The ensuing thematic analysis examines the ways in which temporality – including stakeholder perceptions of the relevance of past practices and planning for an uncertain future - influenced the coordination of the scaling up and adaptation of ICU units.
