# Supplementary material for: “Everything was much more dynamic”: Temporality of health system responses to Covid-19 in Colombia
Source: PLoS One. 2024 Sep 26;19(9):e0311023. doi: 10.1371/journal.pone.0311023 (PMC11426449; doi:10.1371/journal.pone.0311023)
Supplement: S6 Table — (PDF) [file pone.0311023.s007.pdf]

S7 Table. Consequences of the accelerated approach to expanding ICU capacity

**Impact**	**Evidence**
Constrained stakeholder involvement	Perspective of nursing profession neglected:<br><br>"So, life begins to revolve around Covid, but around the situation of the nursing staff in the face of Covid, but also around this situation of indignation as to why the national government only meets with doctors, and what about doctors? What else? If there are many professionals here, well, they are at risk" (SH-A-001, nursing association representative, Bogotá).
Rapid authorisation process	Need for rapid compliance:<br><br>"To find out that this is going to grow and that this is to be prepared and that we have to have our intensive care units ready and prepare for everything, but not stop" (ES-B-003, Hospital director, Bogotá).<br><br>Technical issues:<br>"…in the case of the ICUs, it is a completely new process… initially, the requests entered an email and from there they were managed. Obviously that overwhelmed capacity, at some point we had 800 backlogged requests. (...) that email was changed to a platform, at this time each [clinic] and [insurer] has access to the platform, creates the case, the intensive care unit request, and we generate an authorization" (SH-B-018, Representative, commissioning organization, Bogotá).
Financial impact on providers	"It is that the sense of infrastructure let's say that it is there, the big problem is how to maintain it because you have no income; So, from one moment to another, the hospital that lives by selling services stops selling services, but it has to be prepared for a pandemic" (ES-B-003, Hospital director, Bogotá).
Importance of administrative support	"What else helped us land the situation? The availability of the administrative team of the institution, I think there was an openness from the beginning and that made it easier for

	things to happen. If they had not had the focus, I think we would have worn ourselves out more and would not have managed to get to that point so quickly, but would have taken longer […] everything was much more dynamic, those are like the advantages" (SH-C-021, hospital nurse, Cali).
