# Supplementary material for: “Everything was much more dynamic”: Temporality of health system responses to Covid-19 in Colombia
Source: PLoS One. 2024 Sep 26;19(9):e0311023. doi: 10.1371/journal.pone.0311023 (PMC11426449; doi:10.1371/journal.pone.0311023)
Supplement: S7 Table — (PDF) [file pone.0311023.s008.pdf]

S8 Table. Summary of Findings

	Case study	
**Theme**	**Laboratories**	**Intensive care units (ICUs)**
Temporal urgency	• Rapid adaptation demanded and celebrated by senior level planners and providers of laboratory services.<br><br>• While strategic decisions were taken rapidly, there was less consideration of organizational support for implementation.	• Top-down change, e.g. a legal declaration to increase ICU capacity, stimulated activity at lower levels (e.g. reviewing new protocols).<br><br>• Stakeholders expressed concern about reaching the limits of ICU capacity; the projected threat to life of this future state was described as a "fear" that induced collaboration.
Historical learning	• The past was enacted in particular ways by stakeholders in describing responses to COVID-19. Some stakeholders used the past as a resource to gain influence over the current situation (e.g. describing relevant experiences or skills).<br><br>• The past was also narrated as a locus of failings, signifying a need for new approaches to tasks and relationships during the pandemic.	• Some related experience of emergencies, e.g. earthquakes, informed local planning responses to COVID-19.<br><br>• However, responses were mostly characterised by a lack of relevant experience and were therefore associated with uncertainty and trepidation.
