# Supplementary material for: “Everything was much more dynamic”: Temporality of health system responses to Covid-19 in Colombia
Source: PLoS One. 2024 Sep 26;19(9):e0311023. doi: 10.1371/journal.pone.0311023 (PMC11426449; doi:10.1371/journal.pone.0311023)
Supplement: Attachment — Submitted filename: Response to Reviewers.docx [file pone.0311023.s009.docx]

Dear Dr Pruller,

Many thanks for your decision letter on our manuscript. We are very grateful for your constructive comments and for those of the reviewer. We have made a point-by-point response to all comments in the table below.

Best wishes,

Simon Turner (on behalf of co-authors)

| Editor |  |
| --- | --- |
| The reviewer has recommended that you expand your literature review, and discuss your findings in relation to previous studies about the COVID-19 pandemic. Could you please carefully revise the manuscript to address all comments raised? | Thank you for your steer on addressing the reviewer´s recommendations. We have responded to each comment in turn (see below), which includes improving the literature review and discussion sections of the paper. We have submitted two versions of the paper, with and without tracked changes. |
| Financial disclosure  If you would like to make changes to your financial disclosure, please include your updated statement in your cover letter. | The financial disclosure should be as follows:  “Simon Turner is grateful for funding from the Ministerio de Ciencia Tecnología e Innovación (Mincincias), grant award number 1204101577001, and “Fondo de Apoyo a Profesores Asistentes” (FAPA) de la Universidad de los Andes. The funders had no role in study design, data collection and analysis, decision to publish, or preparation of the manuscript.” |
| Style requirements  Please ensure that your manuscript meets PLOS ONE's style requirements, including those for file naming. The PLOS ONE style templates can be found at  <https://journals.plos.org/plosone/s/file?id=wjVg/PLOSOne_formatting_sample_main_body.pdf> and  <https://journals.plos.org/plosone/s/file?id=ba62/PLOSOne_formatting_sample_title_authors_affiliations.pdf> | We have revised the style of the headings and references in line with the guidance. |
| Copyediting  We suggest you thoroughly copyedit your manuscript for language usage, spelling, and grammar. If you do not know anyone who can help you do this, you may wish to consider employing a professional scientific editing service. Upon resubmission, please provide the following: The name of the colleague or the details of the professional service that edited your manuscript. | Catherine Turner proofread the manuscript and made suggestions for copyediting. |
| Data repository  Note from Emily Chenette, Editor in Chief of PLOS ONE, and Iain Hrynaszkiewicz, Director of Open Research Solutions at PLOS: Did you know that depositing data in a repository is associated with up to a 25% citation advantage (<https://doi.org/10.1371/journal.pone.0230416>)? If you’ve not already done so, consider depositing your raw data in a repository to ensure your work is read, appreciated and cited by the largest possible audience. You’ll also earn an Accessible Data icon on your published paper if you deposit your data in any participating repository (<https://plos.org/open-science/open-data/#accessible-data>). | Please see data availability information below. |
| Funding information  We note that the grant information you provided in the ‘Funding Information’ and ‘Financial Disclosure’ sections do not match. When you resubmit, please ensure that you provide the correct grant numbers for the awards you received for your study in the ‘Funding Information’ section. | The following funding statement should be added to the paper:    “Simon Turner is grateful for funding from the Ministerio de Ciencia Tecnología e Innovación (Mincincias), grant award number 1204101577001, and “Fondo de Apoyo a Profesores Asistentes” (FAPA) de la Universidad de los Andes. The funders had no role in study design, data collection and analysis, decision to publish, or preparation of the manuscript.” |
| Data availability  We note that you have indicated that there are restrictions to data sharing for this study. For studies involving human research participant data or other sensitive data, we encourage authors to share de-identified or anonymized data. However, when data cannot be publicly shared for ethical reasons, we allow authors to make their data sets available upon request. For information on unacceptable data access restrictions, please see <http://journals.plos.org/plosone/s/data-availability#loc-unacceptable-data-access-restrictions>.  Before we proceed with your manuscript, please address the following prompts:  a) If there are ethical or legal restrictions on sharing a de-identified data set, please explain them in detail (e.g., data contain potentially identifying or sensitive patient information, data are owned by a third-party organization, etc.) and who has imposed them (e.g., a Research Ethics Committee or Institutional Review Board, etc.). Please also provide contact information for a data access committee, ethics committee, or other institutional body to which data requests may be sent.  b) If there are no restrictions, please upload the minimal anonymized data set necessary to replicate your study findings to a stable, public repository and provide us with the relevant URLs, DOIs, or accession numbers. Please see <http://www.bmj.com/content/340/bmj.c181.long> for guidelines on how to de-identify and prepare clinical data for publication. For a list of recommended repositories, please see <https://journals.plos.org/plosone/s/recommended-repositories>. You also have the option of uploading the data as Supporting Information files, but we would recommend depositing data directly to a data repository if possible.  Please update your Data Availability statement in the submission form accordingly. | The data availability statement has been updated as follows:  “This paper draws on a qualitative dataset that involved interviews with health system stakeholders involved in the response to the Covid-19 pandemic in Colombia, who represent potentially vulnerable staff at various system levels. The raw interview recordings and transcripts include potentially identifying or sensitive personal and organizational information. Following research ethics guidance relating to participant anonymity, we are not able to disclose the raw data. However, the minimal dataset, with regard to types of interview participant and relevant quotations derived from the interviews, are included in anonymised form in the manuscript. All relevant data are within the manuscript and its Supporting Information files.” |
| Please include captions for your Supporting Information files at the end of your manuscript, and update any in-text citations to match accordingly. Please see our Supporting Information guidelines for more information: <http://journals.plos.org/plosone/s/supporting-information>. | We have moved three tables into the supporting files section and labelled and referenced these appropriately at the end of the manuscript. |
| Reviewer 1 | Response |
| The paper discusses an interesting topic: the role of temporality in crisis responses, through a qualitative analysis of health system providers and stakeholders during the COVID-19 pandemic in Colombia. However, some changes are needed to improve its contributions. | We thank the reviewer for this comment and for their constructive feedback (our point-by-point response is detailed below). |
| First, the paper adopts a specific theoretical discussion related to organizational studies, and some gaps are pointed out, but other approaches, such as historical institutionalism, discuss these gaps (the relevance of external environmental shocks and the importance of past experiences). | We have added studies informed by historical institutionalism to the literature review. We have explained our particular focus on social constructivism in the current paper, but set out how the combination of the two is potentially valuable in the discussion, adding the following text:  “Other theoretical perspectives, notably historical institutionalism, can be applied to the study of temporal change. For example, historical institutionalism recognises tensions between past arrangements, due to path dependency, and path-bending forces associated with external environmental shocks or “critical junctures” [15]. For historical institutionalists, the pandemic represented a window of “political openness” for accelerated governmental decision-making on policy change [16], although the consensus appears to be that radical change was moderated by maintenance of the existing institutional context, including structural interests [17], continuity in the dominant ideological or policy paradigm [18], and flexible use of existing systems [19]. The evidence suggests gradual evolution of existing policy ideas to meet identified public policy challenges of the pandemic rather than a radical disjuncture associated with the Covid-19 crisis [20-22].  “These factors are relevant to the social constructivist perspective adopted in this paper, particularly in highlighting the importance of the macro public policy context in shaping responses to crises like the pandemic, but the approach adopted in this study is limited to analysing the role of temporal resources in meso and micro level responses to the macro level context. Using historical institutionalism to analyse macro level policy actions is beyond the scope of the current study, although we advocate in the discussion for its combination with social constructivism in future research.” (pp.8-9). |
| Secondly, the paper should develop the findings of previous studies about the COVID-19 pandemic, including the COVID-19 pandemic in Colombia. | We have drawn on additional Covid-19 studies in the literature review and explained how the current paper builds on these, adding the following text:  “Key organizational factors in Colombia´s response to the pandemic identified in previous literature include shared purpose that propelled formal [29] and informal collaboration within the health system [30], inter-sectoral collaboration at the local system level [31-32], and repurposing organizational and professional roles to redirect resources toward meeting the emergent strategic goals outlined [33]. This paper builds on these findings by using micro level concepts (the enactment of temporal urgency and historical learning) to analyse how professional and organizational interactions were motivated and negotiated in service of the rapid health system response to the pandemic across two service areas in Colombia. This approach complements previous studies by describing interactional mechanisms through which rapid changes were achieved, while also tracing unintended consequences of the accelerated approach to change, using micro level concepts that capture how and why the temporal context of the pandemic influenced change processes.” (pp.9-10). |
| Thirdly, the study briefly discusses the features of public policy (including the dynamics and legacies of Colombia's health care policy) and the role of politics and political leadership. It would be important to develop that as it would support the case contextualization and the variables that could influence the changes analyzed. | We developed the discussion significantly, adding more detail in the areas of policy, hierarchy, and power, in relation to social constructivism, and signalling where further research could be undertaken at the interface with historical institutionalism. The following text has been added:  “This finding speaks to concerns among social constructivists about using concepts of power to explain the temporal structuring of social interactions [3]. In the critical tasks studied in this paper, leaders´ ability to mobilise temporal resources relied on hierarchical power structures associated with traditional authority and accountability relationships. Temporary temporal resources combined with long-standing hierarchical authority to support accelerated task delegation. Rather than constraining shifts in the temporal structure of tasks as predicted, powerful stakeholder interests supported accelerated change in the approaches to the two tasks we studied. The communication of temporal urgency became an additional tool for maintaining existing managerial and institutional interests in early responses to the pandemic, when seen as a critical juncture. For example, the expansion of ICU capacity was coordinated top-down via a legal decree that set out a national target for expansion, changes to financing, and prescribed organizational roles.  The consequent social interactions around implementing the decree were then characterised by (a) the assertion of urgency by mid-level organizational leaders based on the top-down call for accelerated change, (b) temporally pressured forms of working and organizing and (c) rounds of reinterpretation work at the operational level to align policy dictates with clinical realities. This study contributes to social constructivist accounts of institutional change by showing the importance of relating improvised social actions observable at the micro level to durable forces at the organizational and system levels. These durable forces “power up” leaders´ coordination of resources, in this case temporal ones, to guide the social interactions in and through which critical tasks are accomplished.  Social constructivists need to stitch managerial and institutional power bases into their analysis of social interactions to explain the maintenance of temporal structuring as well as temporal restructuring of task coordination. One promising method for doing this in future studies is to combine insights from macro historical institutionalism with micro level social constructivism. For example, deployment of temporal resources at the micro and meso levels may play a role in shaping policy responses at “critical junctures” like the Covid-19 pandemic. In this study, temporal resources appeared to have a moderating effect on new approaches to change – it was accelerated, but not more egalitarian at the juncture we studied – suggesting that use of temporal resources by actors at the micro level (e.g. communication of urgency in task delegation) maintained existing institutional interests.  Use of past experiences, including reactivation of latent personal networks to share information and resources, were a key temporal resource that supported improvised actions at the operational level to translate top-down directives into practice. Notwithstanding the value of such improvised actions as a source of informal resource coordination, they need to be set against a background of policy and organizational pressures for accomplishing Covid-related tasks. At the operational level, temporal resources were deployed within, and helped predominantly to reproduce rather than challenge, existing hierarchies of power that mediated the translation of initiatives from the policy to operational levels. The servicing of existing hierarchical systems allowed leaders to engage in rapid task delegation and to raise the tempo of work across hierarchical tiers. Organizational hierarchy, and the exercise of positional power this allows in coordinating task and authority relationships, is an additional moderating mechanism for explaining continuity in institutional interests at critical junctures like pandemics.” (pp33-35). |
| Finally, it would be important to better grasp the contributions of the paper to understand other crises or the pandemic in other countries. | Empirical findings. We have added a concluding section to both the laboratory and ICU cases called “Consequences of the accelerated approach to change” to better define the impact of the approach to change in each case and facilitate a better transition inot the discussion where we compare the cases. For the laboratory case, see pages 19-21. For the ICU case, see pages 26-28.  Discussion section. We have spelled out the contributions of the paper, particularly with regard to policy and practice, through the addition of five points to the discussion section of the paper, as follows:  “For future pandemics, the experiential knowledge embodied in mid- and operational-level staff should be drawn upon more fully “upstream” as knowledge inputs or capabilities for enhancing emergency planning and preparedness.” (p.29)  “For future pandemics, lessons drawn from the gradual routinisation of both critical tasks navigated during the Covid-19 pandemic should be incorporated into system-wide learning.” (p.30)  “Patient and public involvement mechanisms need to be carefully planned and structured in advance – taking into account potential imbalances in power, diversity, and social inequities [49] – to regulate the mix of actors informing “co-production” in times of temporal stress. Temporal concepts also point to the value of bringing a variety of past and current experiences to bear on complex problems characterised by uncertainty and time pressure.” (p.36)  “Barriers and enablers to durable organizational learning from pandemics – that involves the translation of experience into standardised routines for guiding action and ongoing feedback and evaluation [50] – should be analysed in specific organizational settings by practitioners and researchers alike.” (p.37)  “More “open” and empathetic forms of leadership [1] – based on acknowledging the human value of, and navigating tasks in close coordination with, mid- and operational- level staff – is one way of countering negative effects upon staff in periods of temporal stress.” (p.37-38)  Reflecting the updated discussion, we have also signposted an additional contribution of the paper in the introduction, as follows:  “Finally, we argue that the deployment of temporal resources maintained, rather than challenged, existing institutional interests in Colombia as the critical Covid-related tasks we studied were assigned and managed via existing organizational hierarchies.” (p.3). |
