# Supplementary material for: “Everything was much more dynamic”: Temporality of health system responses to Covid-19 in Colombia
Source: PLoS One. 2024 Sep 26;19(9):e0311023. doi: 10.1371/journal.pone.0311023 (PMC11426449; doi:10.1371/journal.pone.0311023)
Supplement: Attachment — Submitted filename: Response to Reviewers.docx [file pone.0311023.s010.docx]

6 September 2024

Dear Dr Amer,

“Everything was much more dynamic”: temporality of health system responses to Covid-19 in Colombia

Thank you for your decision letter on our manuscript. The points made have been very useful for improving the readability of the manuscript and improving the methods and discussion sections in particular; we detail in the table how we have responded to each point and include a tracked changes version of the manuscript.

Yours sincerely,

Dr Simon Turner (on behalf of co-authors)

| Journal Requirements |  |
| --- | --- |
| Please review your reference list to ensure that it is complete and correct. If you have cited papers that have been retracted, please include the rationale for doing so in the manuscript text, or remove these references and replace them with relevant current references. Any changes to the reference list should be mentioned in the rebuttal letter that accompanies your revised manuscript. If you need to cite a retracted article, indicate the article’s retracted status in the References list and also include a citation and full reference for the retraction notice. | The reference list has been reviewed and no changes were deemed necessary. |
| Additional Editor Comments: |  |
| The abstract and introduction clearly outline the study’s objectives and significance. The comparative case study approach provides valuable insights into crisis management during the COVID-19 pandemic. The discussion integrates findings with existing literature and highlights the importance of temporality in health system responses. | Thank you for this comment. |
| Length and Redundancy: The manuscript is notably lengthy, and some sections contain redundant content. We recommend revising these sections to make the text more concise. Please utilize the appendices more efficiently to manage detailed content. | The case narratives have been moved to the appendices to make the main text more concise (see S4 and S6 Tables).  Moreover, the consequences of the accelerated approach to change in each case have been tabulated and moved to the appendix (see S5 and S7 Tables).  Through various edits, especially to the literature review, findings, and discussion, the length of the paper has been reduced from 12,894 to 9,118 words (the main text is now 7,243 words). |
| Methods: Provide more clarity on case selection criteria and interview methodology. Expand on how data analysis was conducted, including coding and categorization of data. | We justify the choice of cases as follows:  “We chose two cases, the scaling up of laboratory testing and transformation of ICU capacity, as these tasks (expanding testing to control spread of Covid-19 and providing intensive care for severely affected patients) represented national and international priorities in response to Covid-19.” (p.9).  We have clarified that the interviews were semi-structured using a topic guide (p.10) and the topic guide has now been included as an additional file (S2 Appendix).  We have clarified the process of data analysis to explain coding and categorisation (that is also represented in a coding tree, S3 Table). The revised description of data analysis is as follows (new text in italics):  *“Given our research interest in stakeholders` use of temporal resources to navigate non-routine tasks, we were attentive to how past experiences and imagined futures were perceived by stakeholders and influenced approaches to pandemic-related activities (laboratory testing and ICU capacity).* The data were analysed in two stages drawing on deductive and inductive approaches [35], whereby manual coding of the data by the two researchers was informed both by organizational literature on temporality (*deduced from existing concepts,* e.g. past and future projections, temporal structuring and uncertainty) and by attending to the unique perceptions and experiences of the participants *(inductive). The process of thematic analysis was as follows.* First, narratives of the processes of adapting two aspects of Colombia´s health system in response to the pandemic were constructed: (1) expanding laboratory testing capacity and (2) creating dedicated ICU spaces for patients with Covid-19. For each process, *we drew on interview data to describe* the problem facing the health system, the key actions taken to address this problem, including those that came due to regulations *drawing on relevant policy documents*, and the provisional outcomes from these actions. Second, the processes involved in navigating the tasks were *extracted from the interview data using two themes*: enacting temporal urgency (*sub-categories;* how the severity of the situation was mobilized, influencing motivation to participate and pragmatic actions taken) and enacting historical learning (*sub-categories;* how past experiences, relationships, and perceived system failings were mobilized to gain influence in the current response). Analytic data saturation was reached when a full account of the temporal process of navigating the two tasks was reached. A coding tree of the themes by task is provided in S3 Table.” (p.10-11). |
| Discussion: Break down long paragraphs and ensure clearer connections between findings and broader policy implications. Offer more detailed and actionable recommendations. | The discussion has been broken down into shorter paragraphs; the length has been reduced to bring greater clarity to the main research and policy implications of the research.  Moreover, clear actions have been added to the three policy implications as follows:  “Stakeholder engagement mechanisms tailored to pandemic use should be planned in advance.” (p.25)  “Health system leaders should ensure experiential learning from the pandemic is codified to inform future emergency planning.” (p.26)  “Health providers´ experiences during Covid-19 should inform emergency planning for future pandemics to mitigate the knowledge, capacity, and emotional burden associated with avoidable ad hoc negotiation of tasks during crises.” (p.27) |
| Clarity and Structure: Shorten long paragraphs in the discussion to enhance readability. Address overly dense text to improve reader engagement. | The text has been carefully proof-read and revised to improve readability. |
